# Barriers to Eye Care for Adults in the United States and Solutions for It: A Literature Review

**DOI:** 10.7759/cureus.59071

**Published:** 2024-04-26

**Authors:** Nicholas Green, Faythe Miller, Deepesh Khanna

**Affiliations:** 1 Public Health, Nova Southeastern University Dr. Kiran C. Patel College of Osteopathic Medicine, Fort Lauderdale, USA; 2 Foundational Sciences, Nova Southeastern University Dr. Kiran C. Patel College of Osteopathic Medicine, Clearwater, USA

**Keywords:** telemedicine, vision and eye screening, health communication, eye healthcare literacy, transport and accessibility, healthcare insurance, eye care services, vision loss, barriers to eye care, blindness prevention

## Abstract

Vision loss and blindness is a significant public health concern that has had a profound impact on various communities in the United States. Both anticipated and unforeseen barriers have been linked to the rising rates of vision loss and blindness in the country.

Extensive research has identified numerous barriers that put many Americans at a disadvantage when trying to seek high-quality eye care services. Not only do the barriers to eye care services create problems for eye health, but also create a poor quality of life. Therefore, understanding and identifying barriers to eye healthcare services is incredibly important. In addition to understanding and identifying barriers, it is also important to identify solutions to the problems created by these barriers.

A systematic review of articles characterizing the barriers to eye care was completed which resulted in the identification of the major barriers that affect Americans. The review of previous research was also used to identify available solutions for problems associated with the barriers to eye care services.

The major barriers identified were cost, insurance, transport and accessibility, eye health care literacy, and communication. Because of the identification of the major barriers, solutions were also identified. Health education and increased vision screenings were found to be the most used forms of solutions by healthcare professionals promoting good eye health. Telemedicine has also been cited as a possible solution to the growing problem of visual impairment and blindness within the American population.

## Introduction and background

Vision loss and blindness, which refer to the temporary or permanent decrease in visual acuity [1], represent significant public health concerns that have deeply affected various communities in the United States. According to findings from a Bayesian Meta-analysis conducted by Flaxman et al. (2021) [2], it was estimated that approximately 7.08 million individuals were living with some form of vision loss or blindness in 2017. With the ongoing increase in rates of vision loss and blindness within the United States, the demand for essential eye care services has grown significantly. Simultaneously, public health officials are diligently working to create and fund the expansion of resources that would ensure uninterrupted access to essential services like eye care services. Conversely, there have been challenges by both anticipated and unforeseen barriers that have presented difficulties throughout this expansion process [3]. 

Unforeseen and anticipated barriers often disrupt the right access to high-quality eye care services. These multifaceted and interconnected barriers have become a growing public health concern within the American healthcare system. According to the World Health Organization (2019) [4], over half of the individuals with vision loss or blindness have visual impairments that could have been prevented or avoided. However, barriers have left many Americans suffering from these avoidable visual impairments. Conditions like glaucoma, a disease characterized by blind spots, blindness, and loss of peripheral vision [5], can be positively affected by early detection, tailored treatment, and adherence to therapy [6]. However, socioeconomic barriers can place limitations on accessing early-term medical intervention. Barriers like cost, transport and accessibility, eye health care literacy, and doctor-patient communication can have detrimental effects on visual acuity and, consequently, a patient's quality of life (QOL). 

If patients are unable to afford costs associated with eye exams and eyeglasses or face challenges in accessing their eye care service provider due to distance [3], their QOL will likely be negatively affected. The development of any form of visual impairment can result in patients becoming vulnerable to chronic health conditions, death, falls and injuries, and mental health disorders [7]. This decrease in the patient's quality of life should ignite the desire for solutions to address the mounting problems surrounding barriers to eye care services. 

Public health officials should actively strive to identify remedies to barriers associated with eye care services. Initiatives like the Detroit Vision Project, Manhattan Vision Screening, and the Philly Glaucoma Detection and Treatment Project have placed a strong emphasis on education. The prioritization of health education is incredibly important. Studies show that glaucoma patients with low or poor health literacy have been associated with poor medication adherence and worsening visual field [8]. In addition to health education, public health officials created resources like the Manhattan Screening Project to promote vision screenings. Vision screenings in community settings can have a direct impact on the prevalence of vision loss and blindness as they allow patients and doctors to identify the early beginnings of diseases like glaucoma, diabetic retinopathy, and macular degeneration [9]. Therefore, the objective of this analysis is to understand and characterize the obstacles encountered by patients in accessing eye care services, as determined by existing literature. In addition to identifying barriers, there will also be an exploration of the corresponding solutions.

## Review

Methods 

**Figure 1:**
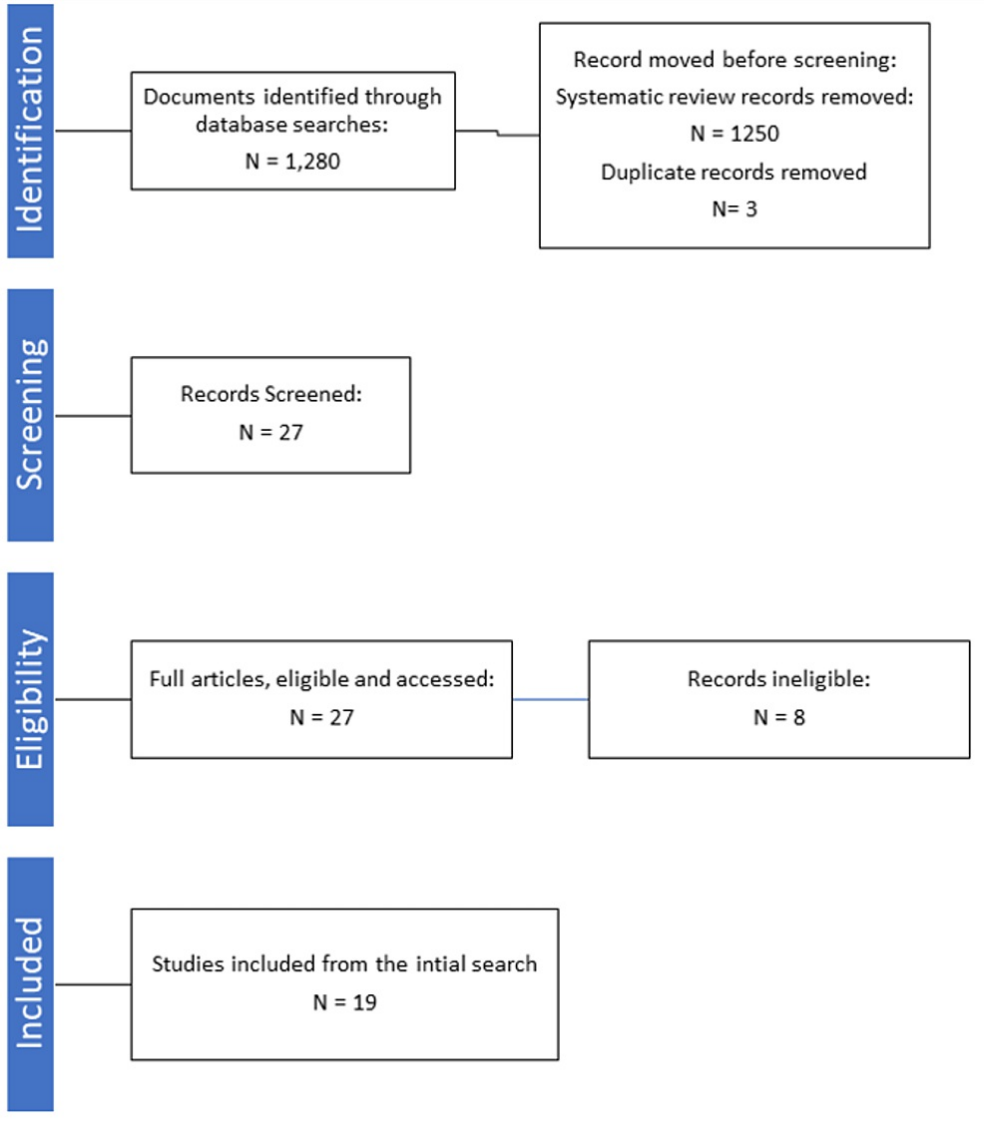
PRISMA flow chart PRISMA - Preferred Reporting Items for Systematic Reviews and Meta-Analysis (PRISMA)

To identify and characterize barriers to eye care in adults, a narrative review was completed using the scholarly databases Google Scholar and PubMed. To locate data, phrases like "barriers to eye care", "barriers to eye exams", "barriers to vision care," and "barriers to eye care solutions" were searched. We considered articles to be eligible if they contained methods and results retrieved from primary research. Articles were also considered if they mentioned barriers to receiving care for specific eye diseases such as diabetic retinopathy and glaucoma or if they mentioned receiving care for specific services such as for low vision care. Research on pediatric eye care was not considered. Articles containing secondary research data were omitted. Articles that focused on a global perspective were also omitted. All articles used had free access to the complete articles either to everyone or to Nova Southeastern University faculty, staff, students, and members of the community. In total, we were left with nineteen articles to complete our analysis. Figure 1 depicts the study selection process. 

Results 

Based on the literature findings, we found four significant reasons for difficulties in patients obtaining eye care with associated solutions. 

Cost and Insurance 

The costs, both with and without insurance, are frequently cited as barriers to receiving care. A cross-sectional survey done by Atta et al. (2022) [10] found that approximately 78% of participants cited high medical costs or a lack of insurance as a barrier to eye-care services. Many patients do have some form of insurance or financial assistance for care. However, out-of-pocket costs like copays, co-insurance, and deductibles may make quality eye care services unaffordable [11]. Most eye care providers require these costs to be paid upfront before care is rendered [3, 12-14]. Even if patients can pay for the exam costs, costs for materials such as eyeglasses, contact lenses, and medications may make treatment compliance difficult for patients. Fear of surprise medical bills long after care is rendered is also cited as a barrier to utilizing eye care services in approximately 31% of patients surveyed in one study in Oregon [15]. The costs of receiving eye care services are perceived by many as not being important when compared to other costs of living such as food, housing, or other medical needs [14]. 

These costs are further increased by limited coverage of eye care services, particularly those offered to patients on state and or federal health insurance programs [15]. Many of these patients are of lower socioeconomic status, meaning these patients are more sensitive to costs [12]. Patients using state or federal health insurance are less likely to be successful in setting eye care appointments in comparison to patients using private insurance. This can be attributed to providers declining or minimizing available appointments for those patients [16, 17]. In addition to difficulties with scheduling appointments, many patients with state and/or federal health insurance will experience delays or inability to set more than one appointment at a time [12, 17]. These factors together lead to the impression amongst patients that doctors are more concerned about profit than a patient's well-being [3]. 

Transportation and Accessibility 

The ability to get eye exams is also cited as the biggest barrier to receiving eye care. Many participants in one study cited the need to ask other family members to provide their transportation [15, 18]. Inconvenient locations of clinics in rural areas with long distances from patients and limited public transportation options contribute to the problem of accessibility [15].

In one study, patients and eye care providers in a community in Alabama were asked about any difficulties in getting eye care. Accessibility and transportation were cited as the biggest perceived barriers to eye care by both providers and patients. Specific comments by patients included the need to have family members take time off work to provide transportation and the inconvenient locations of eye clinics [19].

Even in urban centers with increased public transportation options, transportation can still play a significant role in whether care is received. In a survey of barriers to receiving eye care for patients in Philadelphia, nearly 14% of participants cited an inability to get transportation, and 3.5% cited the cost of transportation as a barrier to receiving follow-up care. Many of these patients suffered from family and friends-provided transportation that was canceled or public transit strikes that inhibited their ability to go to required appointments [18].

In a similar study in Detroit, transportation was found to be a significant barrier to accessing eye care services in a quarter of patients who participated in a vision screening. An inversely proportional relationship was noted between patients with both low socioeconomic status and low educational attainment along with increased transportation difficulties. Interestingly, these patients also noted that improved transportation options would help remind them to come in for their eye exams [12].

Eye Health Literacy 

Health literacy, or the ability to understand and interpret health information, is cited in studies as a contributing factor in patients developing vision impairment [20]. Many patients perceive any vision changes they experience as normal, particularly as they are aging [16]. Moreover, low general health literacy impedes the patient's understanding of when to follow up with providers and the proper use of medications and prescribed treatments [12, 15, 21, 22]. A screening study that assessed 16,587 patients found that 6.58% of these patients had the highest number of visual impairment [22]. This creates a barrier for patients to improve their vision over time as they are unaware of the proper practices that would change the outcome of the disease. Another study cited a particular lack of education about insurance benefits and the difference between medical and vision insurance as a means of preventing care [15]. These costs of receiving eye care services have been perceived by many as not being as important when compared to other costs of living such as food [16]. These payment processes inhibit patients from accessing quality eye care. 

This problem is particularly acute among African Americans and those of lower socioeconomic status [19]. Other studies suggest that minority groups do understand the importance of eye care services, but that more practical considerations play a larger role in utilizing services. Some correlation has been shown between increasing age and a better understanding of the importance of eye care services [12].

This problem can be more critical to address in those with chronic diseases that can affect eye and vision health, such as diabetes, where more knowledge gaps exist [23]. These diseases require more intensive care that patients may not be aware of. One study found most patients knew diabetes can affect their vision, but many did not receive care due to having no visual symptoms [24]. In another survey conducted, many participants knew how often to get diabetic eye exams but were not aware of the reasons why or symptoms that would prompt them to seek immediate care. Many of the survey participants also felt the diabetes disease burden was too much, and they prioritized systemic management over the ocular management of diabetes [24]. Multiple studies mentioned the excessive number of appointments required to manage diabetes lowered the patient's desire to seek eye care services [16, 24].

Knowledge gaps may not only exist among patients but also among eye care providers themselves. In one survey of optometrists, many providers were unaware of the criteria to refer patients for specialty low-vision services and were found to keep more patients in their care that should have been referred to another provider. This creates a significant problem where patients are prevented from getting proper care for their functional vision [25].

Communication 

Doctor-patient communication has been cited by both eye care providers and patients as a barrier to receiving proper care. In one survey of providers and patients, patients cited difficulties in understanding their conditions and care by their eye care providers as increasing difficulty in getting good eye care [26]. Many patients felt doctors spent an inadequate amount of time discussing their diagnosed conditions. Additionally, many African Americans felt providers were not respectful of them or treated them as the equal partners in care that they wanted to be [16]. Conversely, many eye care providers felt patients did not care about their eye health or, at least, did not play an active role, and felt frustrated with their abilities to get them to follow prescribed care [19, 26]. Many eye care providers do not provide enough disease education for patient preferences [26]. Some evidence suggests that there may be racial bias in these responses as providers felt more frustration among their African-American patients compared to other ethnicities [19].

Much of the communication is directed by the physician as opposed to the patient, which can lead to difficulties. In another study, ophthalmologists were filmed educating patients on eye drop compliance and glaucoma. During these interactions, the ophthalmologist talked for 70% of the time. The questions asked them were generally perfunctory and closed-ended, and assumed there were no problems with the treatment plan. Most worryingly, ophthalmologists only detected 27% of patients who had non-compliance with eye drop usage. This was attributed to both the doctors not asking open-ended questions and a desire to not disappoint the ophthalmologist by the patients [26].

Solutions 

Most solutions have traditionally been focused on educating patients about eye health and providing vision screenings, typically targeted to those of lower socioeconomic status [12, 13, 23]. One intervention focused on providing further education and screenings for those at risk of glaucoma. Many of the participants had ocular pathology, but only approximately 25% of patients who had pathology and responded to the three-month follow-up survey were found to have had follow-up care with an appropriate provider. Many of those who did not make a follow-up appointment cited factors such as forgetfulness, inability to make an appointment, and not sensing a need for the exam due to lack of vision complaints [23].

In another study, vision screenings were offered at senior centers within a specific neighborhood, which utilized trained community health workers. Approximately 78% of patients screened failed due to reduced vision and/or the presence of ocular pathology. These participants were then invited for an exam with an optometrist at the same location later or referred to an ophthalmologist if they had ocular pathology. Over 85% of those scheduled to be seen by an optometrist came to that appointment. However, further follow-up care was not assessed [13].

Another study conducted by Goayl et al. on eye health education was combined with a free screening [12]. This took place at a wide variety of sites, including churches, and local community centers. The education discussed the importance of eye health and its relationship with systemic disease and the blinding nature of many eye diseases. One interesting finding from the study was that there was a marked increase in motivation in seeking primary care once participants knew of the relationship between systemic health and their vision. A free vision and eye health screening took place; however, follow-up rates were poor amongst the participants due to previously discussed barriers [12].

With recognition of poor follow-up after screenings, one study tried to correct this. Participants found to be at risk of glaucoma in a local screening were given vouchers stating the benefits of going to an eye exam, watching videos that included testimonials from patients about the benefits of receiving follow-up care, and how to make and keep further follow-up appointments. These interventions were found to provide a very modest but statistically significant increase in the show rate for follow-up appointments [27].

Telemedicine may also provide another pathway to providing ocular health care to some patients. In one study, fundus imaging and education were conducted on patients presenting to an emergency room for other conditions. These images were then read by an ophthalmologist who provided recommendations on receiving follow-up care. Although 78% of patients reported having a regular relationship with an optometrist or ophthalmologist, 20% of the patients screened had a new diagnosis of diabetic retinopathy. This option may provide an avenue that requires minimal eye care staff to be successful [24].

Limitations 

Limitations of this study include the small sample size of articles used due to the narrow focus on barriers to eye care for adults in the United States, and its interventions. There is likely a significant overlap between barriers to accessing other general healthcare services and eye care services. Additional consideration of pediatric eye care access may present its unique challenges and can add another dimension to this conversation. Another limitation is the use of Google Scholar exclusively for research and the use of open-access articles to find the information. Additional sources of relevant information may be contained in articles requiring payment of subscription fees. However, this article serves as a starting point for future research in understanding and ameliorating barriers to accessing eye care services. 

## Conclusions

Based on our research, patients are unable to access and fully utilize eye care services due to issues with transportation, costs, and issues with communication between patients and providers. Many of these factors are influenced by low socioeconomic status and racial and ethnic inequalities within the United States. However, emphasis should be placed on addressing the practical issues surrounding eye care service access to have the most impact. The interventions discussed in the literature focused on one-time vision screenings which, while effective at detecting vision impairment and eye disease, do little in providing long-term follow-up care for patients who need it. Emphasis should be placed on incorporating eye care services in existing community health centers and other available community structures. Addressing the major factors highlighted in this work would be enough for many patients to get access to a critical service and make for a healthier, clearer country. 
